# Supplementary material for: Predicting visual function by interpreting a neuronal wiring diagram
Source: Nature. 2024 Oct 2;634(8032):113–23. doi: 10.1038/s41586-024-07953-5 (PMC11446822; doi:10.1038/s41586-024-07953-5)
Supplement: Supplementary file 6 — CRF and ERF predictions for individual TmY4 and TmY9 cells. Analogous to Supplementary Data 3, but for TmY target types. Shown are the top four monosynaptic pathways, the strongest pathway passing through each of the top ten intermediary types (ranking from Extended Data Fig. 7), and the trisynaptic pathway Tm1–TmY–Dm3–TmY (see the section entitled Prediction of spatial normalization). [file 41586_2024_7953_MOESM6_ESM.zip › DataS4/TmY9q/720575940609491907.pdf]

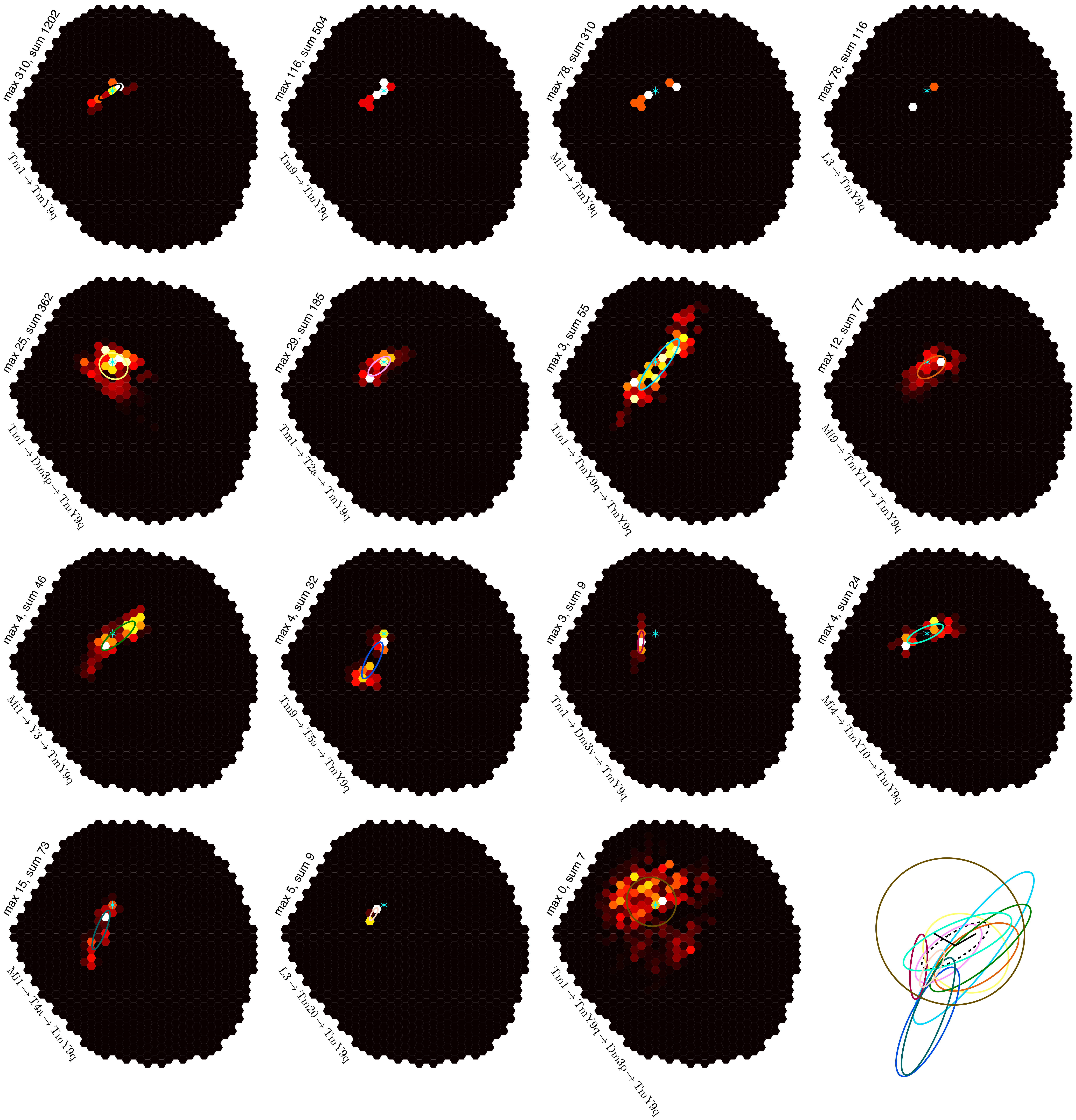

max 310, sum 1202
Tm1 → TmY9q
max 116, sum 504
Tm9 → TmY9q
max 78, sum 310
Mi1 → TmY9q
max 78, sum 116
L3 → TmY9q
max 25, sum 362
Tm1 → Dm3p → TmY9q
max 29, sum 185
Tm1 → T2a → TmY9q
max 3, sum 55
Tm1 → TmY9q → TmY9q
max 12, sum 77
Mi9 → TmY11 → TmY9q
max 4, sum 46
Mi1 → Y3 → TmY9q
max 4, sum 32
Tm9 → T5a → TmY9q
max 3, sum 9
Tm1 → Dm3v → TmY9q
max 4, sum 24
Mi4 → TmY10 → TmY9q
max 15, sum 73
Mi1 → T4a → TmY9q
max 5, sum 9
L3 → Tm20 → TmY9q
max 0, sum 7
Tm1 → TmY9q → Dm3p → TmY9q
